# What are the consequences of ignoring cross-loadings in bifactor models? A simulation study assessing parameter recovery and sensitivity of goodness-of-fit indices

**DOI:** 10.3389/fpsyg.2022.923877

**Published:** 2022-08-18

**Authors:** Carmen Ximénez, Javier Revuelta, Raúl Castañeda

**Affiliations:** ^1^Department of Psychology, Autonoma University of Madrid, Madrid, Spain; ^2^Department of Medicine, Universidad Francisco de Vitoria, Madrid, Spain

**Keywords:** bifactor models, magnitude of factor loadings, cross-loadings, goodness-of-fit, unbiased SRMR index

## Abstract

Bifactor latent models have gained popularity and are widely used to model construct multidimensionality. When adopting a confirmatory approach, a common practice is to assume that all cross-loadings take zero values. This article presents the results of a simulation study exploring the impact of ignoring non-zero cross-loadings on the performance of confirmatory bifactor analysis. The present work contributes to previous research by including study conditions that had not been examined before. For instance, a wider range of values of the factor loadings both for the group factors and the cross-loadings is considered. Parameter recovery is analyzed, but the focus of the study is on assessing the sensitivity of goodness-of-fit indices to detect the model misspecification that involves ignoring non-zero cross-loadings. Several commonly used SEM fit indices are examined: both biased estimators of the fit index (CFI, GFI, and SRMR) and unbiased estimators (RMSEA and SRMR). Results indicated that parameter recovery worsens when ignoring moderate and large cross-loading values and using small sample sizes, and that commonly used SEM fit indices are not useful to detect such model misspecifications. We recommend the use of the unbiased SRMR index with a cutoff value adjusted by the communality level (*R*^2^), as it is the only fit index sensitive to the model misspecification due to ignoring non-zero cross-loadings in the bifactor model. The results of the present study provide insights into modeling cross-loadings in confirmatory bifactor models but also practical recommendations to researchers.

## Introduction

The bifactor measurement model was originally developed by Holzinger and Swineford (1937) to explain hierarchical latent structures of multidimensional variables. It uses a hybrid model which includes a general factor on which all items load and one or more group factors (also known as specific factors) on which subsets of items load. However, the bifactor model was ignored for years and it has been rediscovered as a popular method to model construct dimensionality just in the last two decades (Reise, 2012; Reise et al., 2016). For instance, Cucina and Byle (2017) found that the bifactor model fits better than other latent models to assess the dimensionality of mental abilities test batteries. Moreover, bifactor analysis has been used to model the dimensionality of classical psychological scales (e.g., the Rosenberg Self Esteem scale, Hyland et al., 2014; the Psychopathy Checklist-revised scale, Patrick et al., 2007; and scales of language testing, Dunn and McCray, 2020).

Previous research has examined different issues of the design of the study that affect parameter recovery and the goodness of fit of bifactor models, particularly when an exploratory approach is adopted (Reise, 2012; Morin et al., 2016; Garcia-Garzon et al., 2019; Giordano and Waller, 2020). The present research focuses on the consequences of ignoring cross-loadings in a model when a confirmatory approach is adopted. In confirmatory factor analysis (CFA), it is common to constrain cross-loadings to zero values, assuming therefore that each item loads only on a single construct. However, items are rarely related to a single construct, so this practice may introduce model misspecification and, consequently, a negative impact on the parameter recovery and goodness of fit of the model. Moreover, researchers may not be aware that their models include cross-loadings and therefore to misinterpret their theoretical models. The present study aims to investigate the consequences of ignoring the non-zero cross-loadings in confirmatory bifactor models and evaluate the sensitivity of goodness-of-fit indices to detect such model misspecification.

Previous research has addressed these problems in the context of structural equation modeling (SEM) and CFA models (e.g., Beauducel and Wittmann, 2005; Hsu et al., 2014 and Wei et al., 2022) but few studies have assessed these issues in the context of confirmatory bifactor models (only the recent study by Zhang et al., 2021a). The present study aims to fill this gap and goes beyond previous research in that it focuses specifically on confirmatory bifactor models and considers a wide range of study conditions to assess the importance of the magnitude of the factor loadings in bifactor models. We manipulate the loading size both in the group factors and the cross-loadings. We also study the performance of different goodness-of-fit indices to detect misspecified models by ignoring the non-zero cross-loadings in the group factors. More specifically, our study includes two fit indices for which asymptotically unbiased estimates are implemented in SEM software (the root mean squared error of approximation or RMSEA, and the unbiased standardized root mean squared residual or SRMR*_u_*), and three fit indices in which biased estimators are currently in use (the comparative fit index or CFI, the goodness-of-fit index or GFI, and the SRMR), and we assess their sensitivity to detect misspecified models with the aim of providing practical recommendations to researchers.

The remainder of this article is organized as follows. First, we review previous research on the importance of cross-loadings for parameter recovery of bifactor models. Second, we briefly describe the goodness-of-fit indices used in our study and the way the magnitude of the factor loadings affects them. Next, we describe the design of our simulation study in which we manipulate model specification, sample size, and loading size in the group factors and cross-loadings. We then summarize the results, and evaluate the adequacy of the goodness-of-fit indices to detect model misspecification in bifactor models. We conclude with a general discussion of the results and their practical implications for applied researchers.

## Importance of cross-loadings in bifactor models

In a bifactor model, typically, each item is designed to load on the general factor and on a group factor. However, in practice, items may also have relatively small or moderate non-zero loadings on other group factors, namely cross-loadings. In practical applications, when the bifactor analysis is conducted *via* a confirmatory approach (i.e., CFA), the cross-loadings are fixed at zero values for simplicity and to prevent nonidentification due to approximate linear dependencies between the general factor and the group factors (Zhang et al., 2021b), and nonconvergence, that may arise in bifactor models, particularly if they include large cross-loadings (Mai et al., 2018). However, this practice may result in biased estimates and anomalous results. For instance, forcing even small cross-loadings to zero substantially inflates the estimates of the factor correlations in CFA models (Asparouhov and Muthén, 2009; Mai et al., 2018). Thus, modeling cross-loadings is important, and researchers should be aware that the presence of non-zero cross-loadings does not contaminate the constructs or imply that the fitted model could be inappropriate. On the contrary, constraining non-zero cross-loadings to zero will bias other model parameters (Morin et al., 2016).

The issue of the importance of cross-loadings has been studied in the context of SEM models but it has received less consideration in the context of confirmatory bifactor models. Beauducel and Wittmann (2005) studied the impact of ignoring secondary loadings taking values of 0.20 and 0.40 in CFA models and found that small distortions from simple structure on the data did not lead to misfit in typically used fit indexes (e.g., RMSEA and SRMR). Hsu et al. (2014) considered the impact of ignoring cross-loadings with near-zero magnitudes (0.10 to 0.20 values) in SEM models. They found that the parameter estimates were biased if parameter cross-loadings were higher than 0.13. More specifically, the pattern coefficients and factor covariances were overestimated in the measurement model, whereas the path coefficients with the forced misspecified zero cross-loadings were underestimated. As in the study by Beauducel and Wittmann (2005), they also found that the RMSEA and SRMR indices failed to detect model misspecifications by forcing cross-loadings to zero. More recently, Wei et al. (2022) studied the effect of ignoring cross-loadings in SEM models. They considered cross-loading values ranging from 0 to 0.30 and target loadings ranging from 0.55 to 0.95. They found that the parameter bias was larger as cross-loading values increased and the magnitude of target loadings decreased. However, under conditions of large target loadings (*λ* > 0.80) and medium-large sample size (*N* > 200), the parameter estimation was unbiased.

In the context of confirmatory bifactor models, there is only one recent study (Zhang et al., 2021a) examining the influence of forcing cross-loadings to zero on parameter estimation but it only considers low cross-loadings (values of 0.20 or below). Congruent with the research in CFA models, Zhang et al. (2021a) found that forcing even small cross-loadings to zero leads to biased estimates and large estimation errors of the loadings both in the general factor and in the group factors, such that the loadings in the general factor are overestimated and the loadings in the group factors are underestimated.

The present study aims to analyze the consequences of ignoring non-zero cross-loadings in confirmatory bifactor models. Our study is a follow-up of previous research but it specifically addresses the problem for confirmatory bifactor models and uses a wide range of values for the cross-loadings (near-zero, small, medium, and large) and also for the loadings in the group factors. Parameter recovery is analyzed but the focus of the study is on the usefulness of several goodness-of-fit indices to detect the specification error that involves ignoring small-to-large cross-loadings. The fit indices used in our study are summarized in the next section.

## Effect of the magnitude of factor loadings on goodness-of-fit indices

Another important aim of the present study is to examine the performance of different goodness-of-fit indices to detect the model misspecification when ignoring the non-zero parameter cross-loadings and forcing them to take zero values. Hsu et al. (2014) addressed this issue in the context of SEM models and found that typically used goodness-of-fit indices could not detect the misspecification of forced zero cross-loadings. However, Hsu et al. (2014) considered small parameter cross-loadings (values from 0.07 to 0.19) and only examined the RMSEA and SRMR indices. In the present study, we specifically refer to confirmatory bifactor models and consider a wider range of parameter cross-loading values (from 0.05 to 0.40). Concerning the fit indices, we draw on recent research by Ximénez et al. (2022) to assess the performance of two types of indices: RMSEA and SRMR*_u_*, which are consistent and asymptotically unbiased estimators of the parameter of interest (Maydeu-Olivares, 2017); and CFI, GFI, and SRMR, whose estimators are consistent but are not asymptotically unbiased.

Steiger (1990) pointed out the importance of using unbiased estimators because the sample goodness-of-fit indices can be severely biased at small to moderate sample sizes. The most widely used unbiased index is the RMSEA (Browne and Cudeck, 1993), as it was the first defined at the population level, and it provides a confidence interval for the population parameter and a statistical test of close fit (*H*_0_: RMSEA ≤ *c*). However, its interpretation is problematic because the RMSEA is in an unstandardized metric, and researchers cannot judge whether any given value is large or small (Savalei, 2012). This problem can be avoided using standardized indices (e.g., the SRMR: Jöreskog and Sörbom, 1989). Recently, Maydeu-Olivares (2017) derived an unbiased estimator of the population SRMR (denoted here as SRMR*_u_*), which has shown good statistical properties and efficiency to provide interpretation guidelines to assess the goodness of fit (Shi et al., 2018; Ximénez et al., 2022). Thus, we will use the RMSEA and SRMR*_u_* to represent asymptotically unbiased indices.

Concerning the biased indices, we will refer to the CFI (Bentler, 1990), the GFI (Jöreskog and Sörbom, 1989), and the SRMR index. The CFI and the GFI are relative fit indices and also avoid the interpretation problem of the RMSEA as the fitted model can be compared to an independence model (CFI) or a saturated model (GFI). CFI and GFI are consistent but not asymptotically unbiased indices,^1^ whereas for the SRMR, we will use both the naïve (consistent but biased) sample estimator of the SRMR currently implemented in most SEM software packages and its unbiased estimator (SRMR*_u_*) that is implemented in lavaan (Rosseel, 2012).

Besides illustrating the effect of using biased versus unbiased estimators to detect the model misspecification by ignoring the cross-loadings, the focus of our study is on examining the effect of the magnitude of factor loadings. Previous research has demonstrated that the behavior of fit indices depends on the magnitude of the factor loadings (Steiger, 2000; Saris et al., 2009; Cole and Preacher, 2014). For instance, most fit indices are affected by the phenomenon of the *reliability paradox* or poor measurement quality associated with better model fit (Hancock and Mueller, 2011), such that, as standardized loadings increase, the values of CFI and GFI decrease while the values of RMSEA and SRMR increase. There is a statistical explanation for this phenomenon, and it is that the power of likelihood test statistic depends on the eigenvalues of the model implied covariance matrix, which in turn, depends on the variances of model errors or uniqueness (Browne et al., 2002). Thus, any fit index based on the difference between the observed and implied covariance matrix (e.g., the SRMR) will also depend on the magnitude of the factor loadings (Heene et al., 2011). To avoid the phenomenon of the reliability paradox, Shi et al. (2018) proposed correcting the SRMR value by considering the average communality (*R*^2^) of the observed variables and also a cutoff criterion of $\mathrm{SRMR}/{\bar{R}}^{2}\leq 0.05$ to identify close-fitting models and of $\mathrm{SRMR}/{\bar{R}}^{2}\leq 0.10$ to identify adequate-fitting models. Previous research has found that this correction works well in the context of CFA models (Shi et al., 2018; Ximénez et al., 2022), and our study examines whether the Shi et al.’s (2018) correction works reasonably well to detect misspecified bifactor models.

## Monte Carlo simulation study

We follow the guidelines for Monte Carlo simulation designs in SEM recommended by Skrondal (2000) and Boomsma (2013) to present the design of our simulation study.

### Step 1: Research question and theoretical framework

This research explores the effect that different issues of the design of the study may have on the recovery of factor loadings and the assessment of goodness of fit of confirmatory bifactor models ignoring non-zero cross-loadings in the model specification. The design issues include varying conditions of the magnitude of the loadings in the group factors (*λ*), sample size (*N*), and magnitude of the cross-loadings (*c*), and focuses on the implications of ignoring non-zero cross-loading in the group factors. The effects of these variables on the occurrence of nonconvergent solutions and Heywood cases are also examined as bifactor models are prone to nonconvergent solutions particularly when the cross-loadings are large.

The consequences of ignoring non-zero cross-loadings, forcing them to be zero in the estimated model, have been studied in the context of SEM models. The present study focuses specifically on confirmatory bifactor models and considers a wider range of conditions, not only for the magnitude of the cross-loadings but also for the magnitude of the loadings in the group factors. Additionally, our study analyzes the sensitivity of several goodness-of-fit indices to detect model misspecification. We consider commonly used SEM fit indices such as RMSEA, CFI, GFI, and SRMR (Hu and Bentler, 1999) and also evaluate the performance of the unbiased SRMR index (Maydeu-Olivares, 2017), which has been revealed as the preferred one in recent research (Shi et al., 2018; Ximénez et al., 2022).

The research questions and hypotheses examined are as follows: First, we expect that the parameter recovery will worsen for the misspecified models ignoring the non-zero cross-loadings, and we aim to answer questions such as: Are the loadings in the general factor overestimated? Are the loadings in the group factors underestimated? Are there other conditions of the study design that attenuate these effects? Second, we examine the sensitivity of several goodness-of-fit indices to detect model misspecification to answer questions such as: What is the best goodness-of-fit index? Do I need a specific sample size in my study? Are there other characteristics of the model (e.g., magnitude of factor loadings) that affect the decision on the election of the goodness-of-fit index? Finally, we evaluate whether Shi et al.’s (2018) correction for the SRMR index based on the communality level works reasonably well for detecting misspecified confirmatory bifactor models.

### Step 2: Experimental design

#### Population models

**Figure 1:**
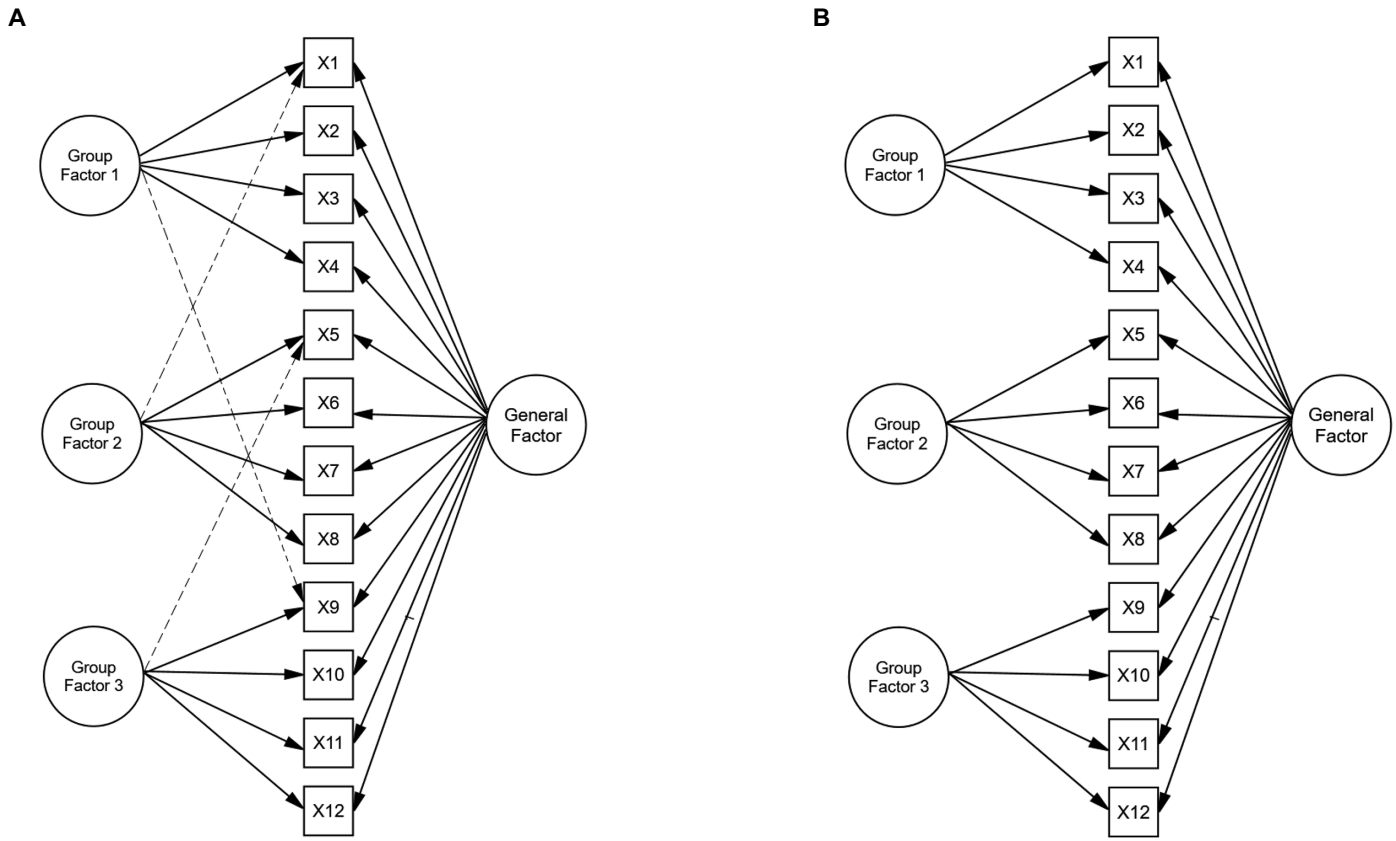
Theoretical and estimated model under the simulation study conditions. **(A)** Theoretical model. **(B)** Estimated model under model misspecification. The three dotted lines in Graph **(A)** are the cross-loadings defined in the theoretical model. Graph **(B)** shows the estimated model under the condition of model misspecification by ignoring the cross-loadings in the theoretical model.

Following Boomsma’s (2013) recommendations, the choice of the population models is based on previous research to increase the comparability of the experimental results and contribute to their external validity. The generating models were defined on the basis of Reise et al.’s (2018) model. More specifically, the population model is a CFA bifactor model with 12 observed variables in which each item depends on a single factor, and there are three group factors, each of which has four indicators (see Figure 1A). As can be seen, the model also includes three cross-loadings, one in each group factor. The loadings in the general factor were fixed to 0.60 to represent strong factor loadings and ensure that a poor recovery of factor loadings was associated with model misspecification. In the group factors, the same number of indicators per factor was used, and the magnitude of the loadings varied between weak (0.15) to strong (0.60) factor loadings. Finally, the magnitude of the cross-loadings varied between nearly zero (0.05) to large (0.40) values.

#### Experimental factors and response variables

The independent variables are the model specification (*q*), the sample size (*N*), the magnitude of the factor loadings on the group factors (*λ*), and the magnitude of the cross-loadings (*c*). Table 1 summarizes the variables used in our design.

**Table 1:** Variables considered in the Monte Carlo study.

Code	Variable	Levels
**Independent variables**		
*q*	Model specification	Correct
		Incorrect (ignoring the cross-loadings)
		
*N*	Sample size	100 (small)
		200 (medium)
		500 (large)
		1,000 (very large)
*λ*	Magnitude of loadings in the group factors	0.15 (very low)
	0.30 (low)
		0.50 (medium)
		0.60 (high)
*c*	Magnitude of cross-loadings	0.05 (near zero)
	0.10 (very low)
		0.20 (low)
		0.30 (medium)
		0.40 (high)
**Dependent variables**		
NCONVER	Nonconvergent solutions (0: Convergent solution; 1: nonconvergent solution)	
HEYWOOD	Heywood cases (0: Non-Heywood case, 1: Heywood case)	
RMSD__FGen_	Recovery of factor loadings for the items of the general factor	
RMSD__FGroup_	Recovery of factor loadings for the items of the group factors	
RMSD__Cross_	Recovery of cross-loadings in the group factors	
GOF	Goodness-of-fit indices: RMSEA, CFI, GFI, SRMR, and SRMR*_u_*	

Model misspecification was introduced by fitting a bifactor model without the cross-loadings to the simulated data. That is, the model was estimated including the cross-loadings (correctly, as in Figure 1A) or ignoring the parameter cross-loadings (incorrectly, as in Figure 1B), forcing them to take zero values.

Sample size included *N* = 100, 200, 500, and 1,000 observations representing small, medium, large, and very large sample sizes. A wide range of sample sizes was used to determine the effect of the magnitude of factor loadings under different conditions of sample size, and to give practical recommendations to researchers about which sample sizes to use to achieve a good parameter recovery and an adequate assessment of the goodness of fit of their models.

The magnitude of the factor loadings in the group factors was specified using four levels: *λ* = 0.15, 0.30, 0.50, and 0.60; representing weak, small, medium, and strong factor loadings (Ximénez, 2006, 2007, 2009, 2016). When generating the data, the loading values used were the same for all variables across factors. The variances of the error terms were set as 1 – *λ*^2^.

The magnitude of the cross-loadings in the group factors was specified using a wide range of levels. Following Gorsuch (1983), cross-loadings up to ± 0.10 are considered random variations from zero. We used five levels: *c* = 0.05, 0.10, 0.20, 0.30, and 0.40, representing almost zero, weak, small, medium, and large cross-loadings, respectively. Given that model misspecification was defined by forcing the cross-loadings to take zero values, these levels also define the degree of model misspecification, which varied between almost null (*c* = 0.05), substantially ignorable (*c* = 0.10), small (*c* = 0.20), medium (*c* = 0.30), and strong (*c* = 0.40).

In summary, the number of conditions examined was 160 = 2 (model specification) × 4 (sample size levels) × 4 (loading levels in the group factors) × 5 (cross-loading levels).

### Step 3: Estimation and replication

For each condition, 1,000 replications were generated with the simsem package in R (Pornprasertmanit et al., 2021). Data were generated from a multivariate normal distribution. Maximum likelihood (ML) estimates of the parameters and goodness-of-fit indices were computed with the *lavaan* package in R (Rosseel, 2012; R Development Core Team, 2019). Parameters were estimated for the models defined in Figure 1. That is, for the correct model, which includes the cross-loadings in the group factors (Figure 1A), and the incorrect model, which ignores such cross-loadings, forcing them to take zero values (Figure 1B).

### Step 4: Analyses of output

Nonconvergent solutions and Heywood cases were deleted to study the effects of the independent variables on the recovery of factor loadings and the goodness of fit. Prior to deleting such solutions, we created two qualitative variables (NCONVER and HEYWOOD) coded as 0 and 1 (see Table 1) and conducted Log-linear/logit analyses to study the effect of the independent variables on the occurrence of nonconvergent solutions and Heywood cases.

The recovery of factor loadings was assessed by examination of the correspondence between the theoretical loading and the estimated one. We used the root mean square deviation or RMSD (Levine, 1977) for each factor in the theoretical model:


(1) $${\mathrm{RMSD}}_{k}=\sqrt{\overset{p}{\underset{i=1}{\sum }}{\left(\lambda_{ik\left(t\right)}-\lambda_{ik\left(e\right)}\right)}^{2}/p,}$$

where *p* is the number of variables that define the factor *k*, *λ*_*ik*(*t*)_ is the theoretical loading for the observed variable *i* on the factor *k*, and *λ*_*ik*(*e*)_ is the corresponding loading obtained from the simulation data. We computed a separate RMSD index for each type of factor loading: one for the 12 loadings in the general factor (RMSD__FGen_), another one for the 12 loadings in the group factors (RMSD__FGroup_), and a final one for the three cross-loadings in the group factors (RMSD__Cross_). The RMSD index defined in Equation 1 is difficult to interpret as its values range between zero (perfect pattern-magnitude match) and two (all loadings are equal to unity but of opposite signs). In practical applications, most studies consider that RMSD values below 0.20 are indicative of a satisfactory recovery (Ximénez, 2006).

A meta-model was used to analyze the results, which included the main effects and the two-, three-, and four-way interaction effects among the independent variables:


(2) $$\begin{array}{c} \mathrm{DV}=\mu +q+N+\lambda +c+q^{*}N+q^{*}\lambda +q^{*}c+N^{*}\lambda +N^{*}c\\ +\lambda^{*}c+q^{*}N^{*}\lambda +q^{*}N^{*}c+q^{*}\lambda^{*}c+N^{*}\lambda^{*}c+q^{*}N^{*}\lambda^{*}c, \end{array}$$

where DV: dependent variables (RMSD and goodness-of-fit measures), *q*: model specification (correct vs. incorrect), *N*: sample size (100, 200, 500, and 1,000). *λ*: loading size in the group factors (0.15, 0.30, 0.50, or 0.60), *c*: magnitude of the cross-loadings (0.05, 0.10, 0.20, 0.30 or 0.40).

A separate analysis of variance (*ANOVA*) was conducted for each of the dependent variables (the three RMSD measures and the five goodness-of-fit values) to test the effects included in the meta-model of Equation (2). For RMSD__Cross_, the meta-model includes all terms except the ones referring to *q* because it only considers correctly specified models. As the large sample size (*n* = 200,000) can cause even negligible effects to be statistically significant, the explained variance associated with each of the effects was also calculated, using the partial eta-squared statistic (*η*^2^). The magnitude of the effects was judged with the interpretation guidelines suggested by Cohen (1988): *η*^2^ values from 0.05 to 0.09 indicate a small effect, from 0.10 to 0.20, a medium effect; and above 0.20, a large effect.

## Results

### Nonconvergence and Heywood cases

The proportion of nonconvergent solutions and Heywood cases that occurred when obtaining 1,000 good solutions per cell is summarized in Supplementary Table S1. Of the 200,000 solutions, 15,297 (7.6%) were nonconvergent and 8,892 (4.4%) presented Heywood cases. The results of the log-linear/logit analyses indicate that the proportion of nonconvergent solutions and Heywood cases was higher when the loadings in the group factors were weak (0.30 or below) and the sample size was decreased. The λ*N interaction effect was of considerable size. Analyses showed that the largest proportion of nonconvergent solutions and Heywood cases occurred for *λ* = 0.15 and *N* = 100 across all the values of the cross-loadings. Furthermore, the proportion of nonconvergent and improper solutions was similar regardless of model misspecification.

Overall, results indicate that the conditions manipulated in our study do not produce a large number of improper solutions and, congruent with recent research (Cooperman and Waller, 2021), nonconvergent solutions and Heywood cases appear only in poorly defined factors (e.g., *λ* < 0.30 in the group factors) and when using very small sample sizes (e.g., *N* = 100).

### Recovery of factor loadings

**Figure 2:**
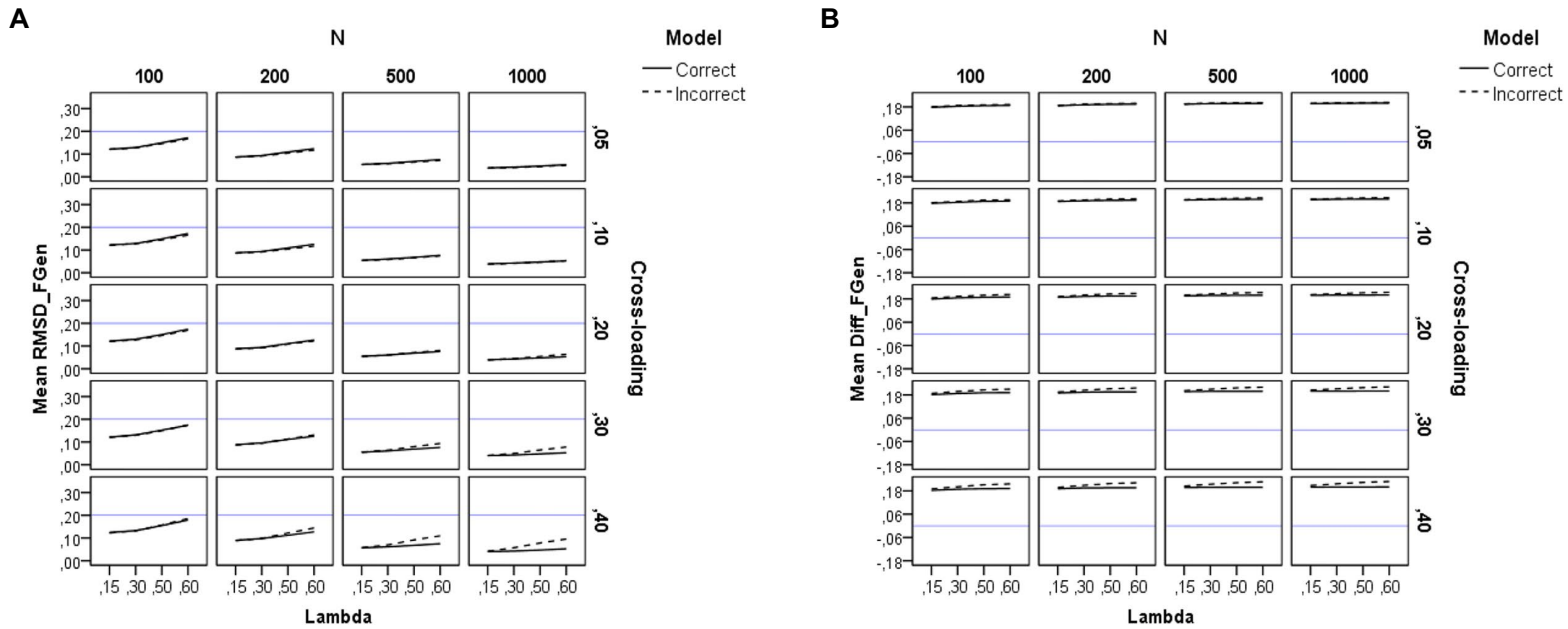
Results for the general factor under the simulation study conditions. **(A)** Recovery of factor loadings in the general factor. **(B)** Difference between estimated and theoretical loadings in the general factor. Model is model specification (correct or incorrect by omitting the cross-loadings), *N* is the sample size (100, 200, 500, and 1,000), Lambda is the magnitude of the loadings in the group factors (0.15, 0.30, 0.50, and 0.60), Cross-loading is the magnitude of the cross-loadings (0.05, 0.10, 0.20, 0.30, and 0.40), and the blue solid line corresponds to the RMSD ≤ 0.20 cutoff in the graph **(A)** and to the null difference (Diff) between the theoretical and the empirical loadings in the graph **(B)**.

**Figure 3:**
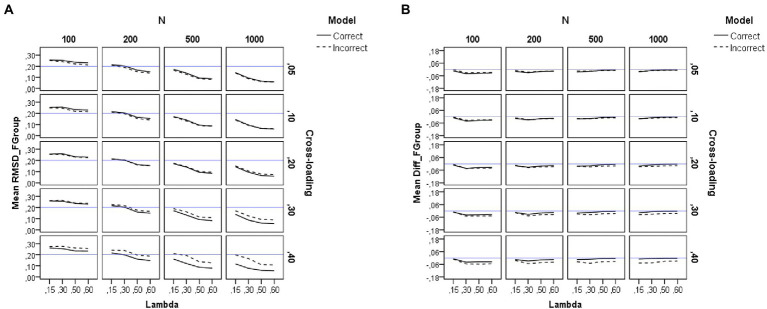
Results for the group factors under the simulation study conditions. **(A)** Recovery of factor loadings in the group factors. **(B)** Difference between estimated and theoretical loadings in the group factors. Model is model specification (correct or incorrect by omitting the cross-loadings), *N* is the sample size (100, 200, 500, and 1,000), Lambda is the magnitude of the loadings in the group factors (0.15, 0.30, 0.50, and 0.60), Cross-loading is the magnitude of the cross-loadings (0.05, 0.10, 0.20, 0.30, and 0.40), and the blue solid line corresponds to the RMSD ≤ 0.20 cutoff in the graph **(A)** and to the null difference (Diff) between the theoretical and the empirical loadings in the graph **(B)**.

**Figure 4:**
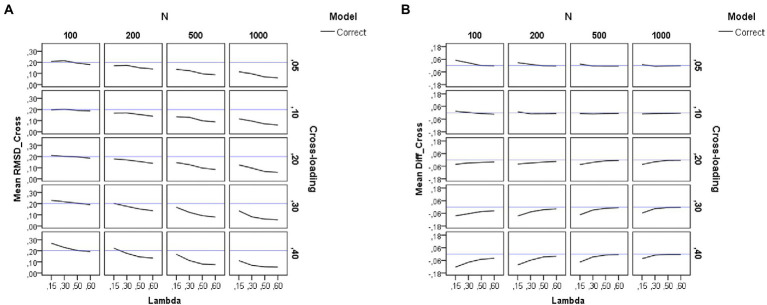
Results for the cross-loadings in the group factors under the simulation study conditions. **(A)** Recovery of cross-loadings in the group factors. **(B)** Difference between estimated and theoretical cross-loadings. Model is model specification (correct or incorrect by omitting the cross-loadings), *N* is the sample size (100, 200, 500, and 1,000), Lambda is the magnitude of the loadings in the group factors (0.15, 0.30, 0.50, and 0.60), Cross-loading is the magnitude of the cross-loadings (0.05, 0.10, 0.20, 0.30, and 0.40), and the blue solid line corresponds to the RMSD ≤ 0.20 cutoff in the graph **(A)** and to the null difference (Diff) between the theoretical and the empirical factor loadings in the graph **(B)**.

Table 2 summarizes the results of the *ANOVAs* performed on each dependent variable. Results for the RMSD measures appear in the left-hand side of Table 2. For visual presentations of the patterns, we also plotted in Figures 2A, 3A, 4A the average sample estimates of the RMSD values against model specification (correct or incorrect), loading level in the group factors (*λ* = 0.15, 0.30, 0.50, and 0.60), sample size (*N* = 100, 200, 500, and 1,000), and magnitude of cross-loadings (*cλ* = 0.05, 0.10, 0.20, 0.30, and 0.40). A horizontal blue line has been drawn in these graphs to mark the recommended cutoff value for RMSD (0.20).

**Table 2:** ANOVA results for the effects of the independent variables on the recovery of factor loadings and the goodness of fit.

		RMSD__FGen_		RMSD__FGroup_		RMSD__Cross_		RMSEA		CFI		GFI		SRMR		SRMR*_u_*	
	*df*	*p*	*η* ^2^	*p*	*η* ^2^	*p*	*η* ^2^	*p*	*η* ^2^	*p*	*η* ^2^	*p*	*η* ^2^	*p*	*η* ^2^	*p*	*η* ^2^
*q*	1	<0.001	0.00	<0.001	0.01	–	–	<0.001	0.06	<0.001	0.05	<0.001	0.12	<0.001	0.19	<0.001	0.21
*N*	3	<0.001	0.68	<0.001	0.57	<0.001	0.33	<0.001	0.12	<0.001	0.17	<0.001	0.91	<0.001	0.90	<0.001	0.09
*λ*	3	<0.001	0.22	<0.001	0.22	<0.001	0.09	<0.001	0.03	<0.001	0.01	<0.001	0.03	<0.001	0.00	<0.001	0.07
*c*	4	<0.001	0.02	<0.001	0.02	<0.001	0.00	<0.001	0.05	<0.001	0.04	<0.001	0.05	<0.001	0.09	<0.001	0.08
q*N	3	<0.001	0.01	<0.001	0.01	–	–	<0.001	0.01	<0.001	0.00	<0.001	0.01	<0.001	0.01	<0.001	0.01
q*λ	3	<0.001	0.00	<0.001	0.00	–	–	<0.001	0.02	<0.001	0.02	<0.001	0.02	<0.001	0.06	<0.001	0.05
q*c	4	<0.001	0.02	<0.001	0.04	–	–	<0.001	0.06	<0.001	0.05	<0.001	0.05	<0.001	0.10	<0.001	0.09
N*λ	9	<0.001	0.04	<0.001	0.04	<0.001	0.01	<0.001	0.00	<0.001	0.01	<0.001	0.00	<0.001	0.02	<0.001	0.01
N*c	12	<0.001	0.00	0.002	0.00	<0.001	0.01	<0.001	0.01	<0.001	0.00	<0.001	0.00	<0.001	0.02	<0.001	0.01
λ*c	12	<0.001	0.01	<0.001	0.00	<0.001	0.01	<0.001	0.02	<0.001	0.02	<0.001	0.02	<0.001	0.05	<0.001	0.04
q*N*λ	9	<0.001	0.00	0.011	0.00	–	–	<0.001	0.00	<0.001	0.00	<0.001	0.00	<0.001	0.00	<0.001	0.00
q*N*c	12	<0.001	0.00	<0.001	0.00	–	–	<0.001	0.01	<0.001	0.00	<0.001	0.00	<0.001	0.01	<0.001	0.00
q*λ*c	12	<0.001	0.01	<0.001	0.00	–	–	<0.001	0.02	<0.001	0.02	<0.001	0.02	<0.001	0.04	<0.001	0.03
N*λ*c	36	<0.001	0.00	0.008	0.00	<0.001	0.00	<0.001	0.00	0.413	0.00	0.998	0.00	<0.001	0.00	<0.001	0.00
q*N*λ*c	36	<0.001	0.00	<0.001	0.00	–	–	<0.001	0.00	<0.001	0.00	<0.001	0.00	<0.001	0.00	<0.001	0.00

RMSD__FGen_, RMSD__FGroup_, and RMSD__Cross_ are the RMSD indices for the general factor, the group factors and the cross-loadings, respectively. RMSEA, root mean squared error of approximation; CFI, comparative fit index; GFI, goodness-of-fit index; SRMR, standardized root mean squared residual; SRMR_u_, unbiased SRMR; *df*, degrees of freedom; *p*, value of *p*; *η*^2^, partial eta-squared; *q*, model specification; *N*, sample size; *λ*, magnitude of loadings in the group factors; and *c*, magnitude of cross-loadings.

### Recovery of the loadings in the general factor

As shown on the left-hand side of Table 2, the sample size (*N*) and the magnitude of the loadings in the group factors (*λ*) have a large effect on the recovery of the loadings of the general factor (*η*^2^ = 0.68 and 0.22), whereas their interaction (N*λ) exerts a small effect (*η*^2^ = 0.04). The pattern of the graphs of Figure 2A indicates that the loadings in the general factor are adequately recovered across all the study conditions, even in the models misspecified by ignoring the cross-loadings (for more details, see also Supplementary Table S2). However, the recovery worsens when the sample size is small (e.g., *N* = 100) and the magnitude of the loadings in the group factor increases (e.g., *λ* = 0.60). Therefore, forcing the non-zero parameter cross-loadings to take zero values does not affect the recovery of the loadings in the general factor when using sample sizes of 200 or more observations.

Figure 2B shows the difference between the estimated loadings and the theoretical ones for the items in the general factor. As can be seen, congruent with the results found in previous research, there is a tendency to overestimation of the loadings in the general factor, regardless of sample size. This effect is more pronounced for the incorrect models (dotted lines) and the cross-loadings with larger values.

#### Recovery of the loadings in the group factors

As shown on the left-hand side of Table 2, the sample size (*N*) has a large effect (*η*^2^ = 0.57) on the recovery of the loadings of the group factors. The pattern of the graphs of Figure 3A indicates that the loadings in the group factors are adequately recovered across all the study conditions, even in the models misspecified by ignoring the non-zero cross-loadings, when the sample size is large (*N* = 500 or more observations). However, recovery worsens when the sample size is small (e.g., *N* = 100). The magnitude of the loadings in the group factor also exerts an effect (*η*^2^ = 0.22), as recovery worsens as the loading in the group factor decreases. Thus, forcing the cross-loadings to take zero values only affects the recovery of the loadings in the group factors when using small sample sizes (200 or fewer observations).

Figure 3B shows the difference between the estimated loadings and the theoretical ones for the items in the group factors. As can be seen, the difference is null only for the correct models and large sample sizes (*N* = 500 or more observations). However, the loadings in the group factors are underestimated when the sample size is small (*N* = 200 or less), and this effect is more pronounced for incorrect models with larger values in the cross-loadings (for more details, see also Supplementary Table S3).

Our findings of overestimation of the loadings in the general factor and underestimation of the loadings in the group factors are congruent with previous research (Hsu et al., 2014; Zhang et al., 2021a; Wei et al., 2022). These results also reflect that the phenomenon of *factor collapse* (Geiser et al., 2015; Mansolf and Reise, 2016) may have operated. Factor collapse occurs when an amount of variance is shifted away from one or more group factors toward the general factor, as happens here, given that the loadings in the general factor are inflated when ignoring the non-zero parameter cross-loadings, whereas the values of the loadings in the group factors are decreased.

#### Recovery of the cross-loadings

In this case, model specification is not an experimental condition, as cross-loadings are only estimated for the correct models. As shown on the left-hand side of Table 2, the pattern of results for the recovery of cross-loadings is very similar to the one already commented on for the recovery of the loadings in the group factors. The sample size (*N*) and the magnitude of the loadings in the group factors (*λ*) have a large (*η*^2^ = 0.33) and medium (*η*^2^ = 0.09) effect on the recovery of the cross-loadings. The pattern of the graphs of Figure 4A indicates that the cross-loadings are adequately recovered across all the study conditions, except when the sample size is very small (*N* = 100) and the magnitude of the loadings in the group factor decreases (*λ* < 0.50).

Figure 4B shows the difference between the estimated loadings and the theoretical ones for the cross-loadings in group factors. As can be seen, the difference is null under the conditions of medium and large loadings in the group factors. However, in the models with weak loadings in the group factors, there is a tendency to underestimate the cross-loadings. This effect is more pronounced for smaller sample sizes and larger cross-loadings.

Overall, the finding of underestimation of the loadings both for the group factors and the cross-loadings may explain the bias of overestimation of the loadings in the general factor and how the phenomenon of factor collapse affects the group factors.

### Goodness-of-fit indices

**Figure 5:**
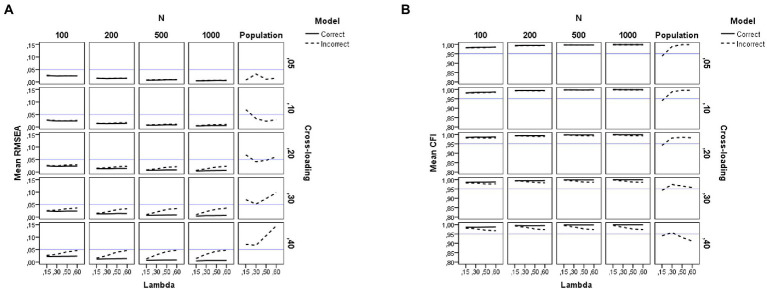
Results for the RMSEA and CFI fit indices under the simulation study conditions. **(A)** RMSEA. **(B)** CFI. Model is model specification (correct or incorrect by omitting the cross-loadings), *N* is the sample size (100, 200, 500, and 1,000), Lambda is the magnitude of the loadings in the group factors (0.15, 0.30, 0.50, and 0.60), Cross-loading is the magnitude of the cross-loadings (0.05, 0.10, 0.20, 0.30, and 0.40), Population is the population values for each index, and the blue solid line refers to the corresponding cutoff for the goodness-of-fit index.

**Figure 6:**
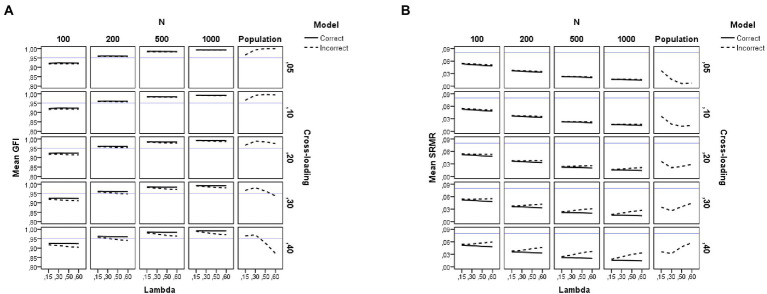
Results for the GFI and SRMR fit indices under the simulation study conditions. **(A)** GFI. **(B)** SRMR. Model is model specification (correct or incorrect by omitting the cross-loadings), *N* is the sample size (100, 200, 500, and 1,000), Lambda is the magnitude of the loadings in the group factors (0.15, 0.30, 0.50, and 0.60), Cross-loading is the magnitude of the cross-loadings (0.05, 0.10, 0.20, 0.30, and 0.40), Population is the population values for each index, and the blue solid line refers to the corresponding cutoff for the goodness-of-fit index.

**Figure 7:**
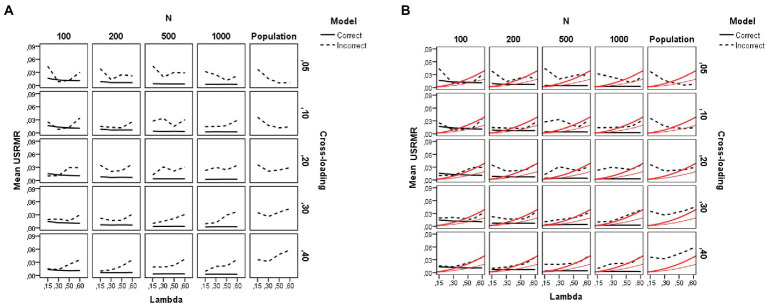
Results for the unbiased SRMR fit indices under the simulation study conditions. **(A)** *SRMR_u_*. **(B)** ${\mathrm{SRMR}}_{u}/{\bar{R}}^{2}$ . Model is model specification (correct or incorrect by omitting the cross-loadings), *N* is the sample size (100, 200, 500, and 1,000), Lambda is the magnitude of the loadings in the group factors (0.15, 0.30, 0.50, and 0.60), Cross-loading is the magnitude of the cross-loadings (0.05, 0.10, 0.20, 0.30, and 0.40), and Population is the population values for each index. The black lines reflect the behavior of the sample *SRMR_u_* under the study conditions (solid line is for the correct model and dotted line for the incorrect model), and the red solid lines show the two cutoffs for ${\mathrm{SRMR}}_{u}/{\bar{R}}^{2}$ : 0.05 (red thin line, close fit) and 0.10 (red thick line, adequate fit).

The right-hand side of Table 2 summarizes the results of the *ANOVAs* performed on each of the goodness-of-fit indices considered here (the descriptive statistics for all fit indices are summarized in Supplementary Tables S4–S6). For visual presentations of the patterns, we plotted the average sample estimates of the fit indices against the simulation study conditions in Figures 5–7. Each figure includes an additional column with the population value for each fit index under the incorrect model. Moreover, a horizontal blue line has been drawn in these figures to mark the recommended cutoff values (Hu and Bentler, 1999) for RMSEA (0.05), CFI (0.95), GFI (0.95), and SRMR (0.08). Below, we summarize the main findings for each fit index.

#### RMSEA results

As seen in Table 2, the largest effects found in the *ANOVA* for RMSEA are the sample size (*N*, *η*^2^ = 0.12), model specification (*q*, *η*^2^ = 0.06), magnitude of cross-loadings (*c*, *η*^2^ = 0.05) and their interaction (q*c, *η*^2^ = 0.06) but these effects are moderate or small. The graphs of Figure 5A show that the RMSEA value is larger for the misspecified models but this effect depends on the sample size and the magnitude of the loadings in the group factors. The use of Hu and Bentler’s (1999) cutoff (RMSEA < 0.05) will lead us to conclude that all conditions provide a close fit to the estimated model when the cross-loadings adopt low values (*c* ≤ 0.20). However, the fit is poorer (sample RMSEA values between 0.07 and 0.11) when the model is misspecified by ignoring medium and large cross-loadings (*c* > 0.20) and the magnitude of the loadings in the group factors is large (*λ* > 0.50).

The graphs of Figure 5A also reflect the difference between the population RMSEA values and the estimated ones. As can be seen, the RMSEA value is underestimated under all the study conditions. However, this effect is more pronounced for the models with larger cross-loadings values (*c* = 0.30 and 0.40), indicating that the model misspecification is not detected by the RMSEA index, regardless of sample size. This result is important as applied studies have found such a pattern in bifactor models (see, for instance, Hörz-Sagstetter et al., 2021).

Our finding of RMSEA not being useful to detect model misspecification when ignoring cross-loadings with near-zero or lower values is congruent with Hsu et al.’s (2014) study, not being particularly detrimental, as the effect size of model misfit for these conditions is negligible. However, under the conditions of large cross-loadings, the effect size of the misfit is considerable and the RMSEA index is unable to detect and reject the model.

#### Comparative fit index results

Table 2 and Figure 5B show the results of the *ANOVA* for the sample estimates of CFI as a function of model specification (*q*), sample size (*N*), magnitude of loadings in the group factors (*λ*), and magnitude of cross-loadings (*c*). As seen in Table 2, for CFI, the pattern of results is similar to the one already commented on for RMSEA. The largest effects found are due to the sample size (*N*, *η*^2^ = 0.17), model specification (*q*, *η*^2^ = 0.05), magnitude of cross-loadings (*c*, *η*^2^ = 0.04) and their interaction (q*c, *η*^2^ = 0.05) but these effects are moderate or small. As seen in Figure 5B, the pattern of results is similar across all the study conditions. In this case, the use of Hu and Bentler’s (1999) cutoff (CFI > 0.95) will lead us to conclude that there is an adequate fit under all the study conditions, even when the model is misspecified by ignoring the cross-loading values. The only conditions where CFI is a bit poorer (population CFI values between 0.91 and 0.94) occur when the model is misspecified and the magnitude of cross-loadings is large (e.g., *c* = 0.40), but the sample CFI is unable to detect and reject such models.

We therefore conclude that the CFI is not useful to detect model misspecification by constraining to zero the coefficients of the cross-loadings and recommend not assessing bifactor models solely based on the CFI index.

#### Goodness-of-fit results

The results for the GFI replicate those already explained for the CFI. However, the effect found in the *ANOVA* due to sample size is much larger (*N*, *η*^2^ = 0.91) and model specification exerts a medium effect (*q*, *η*^2^ = 0.12). The graphs of Figure 6A show that the values for GFI are indicative of an adequate fit when a large sample size is used (*N* = 500 or more observations), regardless of model misspecification. However, when a small sample size is used (*N* = 200 or fewer observations), the GFI values are indicative of a poorer fit (values between 0.85 and 0.93). For instance, either a correct or an incorrect bifactor model by ignoring the cross-loadings would be rejected using the conventional cutoff with samples of 100 observations.

#### SRMR results

This section summarizes the results for all SRMR indices considered here. Concerning the naïve SRMR index, as shown in the right-hand side of Table 2, the main drivers of the behavior of the SRMR are sample size (*η*^2^ = 0.90) and model specification (*η*^2^ = 0.19). The magnitude of the cross-loadings and its interaction with model specification also have a medium effect on the SRMR index (*η*^2^ = 0.09 for *c* and *η*^2^ = 0.10 for q*c). As with the other biased fit indices (CFI and GFI), the pattern of the graphs of Figure 6B indicates that the use of Hu and Bentler’s (1999) cutoff (SRMR <0.08) will lead us to conclude that all conditions provide a close fit to the estimated model, even in those with small sample sizes.

Concerning the behavior of the unbiased SRMR index (SRMR*_u_*), the pattern of results is quite different. As expected, and congruent with previous research, sample size is a main driver of the behavior of the SRMR (biased) index, whereas the SRMR*_u_* (unbiased) index is barely affected by the number of observations in the sample, as *N* exerts an effect but it is much smaller (*η*^2^ = 0.09). As seen in Figure 7A, for the SRMR*_u_* index, all conditions show acceptable goodness-of-fit values according to Hu and Bentler’s (1999) cutoff (SRMR <0.08) and therefore, the unbiased SRMR index is not sensitive to model misspecification by ignoring the cross-loadings. Concerning the difference between the population SRMR values and the estimated ones, similar as with RMSEA, the SRMR*_u_* value is underestimated in the models with large cross-loadings values (*c* = 0.30 and 0.40).

In addition, both the magnitude of the loadings in the group factors (*λ*) and of the cross-loadings exert a small effect (*η*^2^ = 0.07 and 0.08, respectively) on the SRMR*_u_* index, such that the smaller the factor loading, the better the fit. This result indicates that the reliability paradox may have operated. We then analyzed Shi et al.’s (2018) correction of the SRMR*_u_* index based on communality. Figure 7B illustrates the behavior of Shi et al.’s (2018) correction under the simulation conditions (for more details, see also Supplementary Table S6). This figure is similar to Figure 7A but, in this case, the red lines, instead of representing Hu and Bentler’s (1999) cutoff value (i.e., SRMR <0.08), mark Shi et al.’s cutoff values for close-fit ( ${\mathrm{SRMR}}_{u}/{\bar{R}}^{2}\leq 0.05$ , red thin line) and adequate-fit ( ${\mathrm{SRMR}}_{u}/{\bar{R}}^{2}\leq 0.10$ , red thick line). As can be seen, the black solid lines fall below the cutoffs in most of the conditions, indicating a close-fit for the correct models, whereas the black dotted lines are above such cutoffs in the misspecified models indicating unacceptable goodness of fit for the bifactor models ignoring the non-zero parameter cross-loadings. At the population level, the two Shi et al.’s cutoffs work reasonably well to detect the misspecified models. At the sample level, the result for Shi et al.’s correction is clearer for the ${\mathrm{SRMR}}_{u}/{\bar{R}}^{2}\leq 0.05$ cutoff (close-fitting models), whereas the ${\mathrm{SRMR}}_{u}/{\bar{R}}^{2}\leq 0.10$ cutoff (adequate-fitting models) is only satisfied under conditions of low loadings in the group factors. Finally, and congruent with the findings for the asymptotically unbiased indices (RMSEA and SRMR*_u_*), under conditions of small sample size (e.g., *N* = 100) and weak factor loadings in the group factors (*λ* = 0.30 or below), the goodness of fit is inappropriate even for the correct models.

In summary, our results indicate that the correction proposed by Shi et al. (2018) to determine the close fit of the SRMR index as a function of the communality works reasonably well for detecting misspecified bifactor models ignoring low-to-moderate and high cross-loadings. Shi et al.’s cutoffs detect misspecified models whereas the SRMR*_u_* index without the correction cannot detect and reject a misspecified confirmatory bifactor model that ignores the non-zero cross-loadings.

## Discussion and conclusion

The aim of the present study was to assess the consequences of ignoring non-zero parameter cross-loadings in confirmatory bifactor models. We analyzed the recovery of factor loadings and also studied the sensitivity of several typically used goodness-of-fit indices and their cutoffs to detect model misspecification. Previous research has addressed these issues in the context of SEM models but our research focuses specifically on confirmatory bifactor analysis and includes design variables that had not been considered before. For instance, we manipulated a wide range of values both for the loadings in the group factors and the cross-loadings. Moreover, we analyzed the performance of several goodness-of-fit indices to detect model misspecification.

We presented the results of a simulation study investigating the problem of ignoring the non-zero parameter cross-loadings and how it affects parameter recovery and the goodness of fit of the confirmatory bifactor model under varying conditions of sample size and magnitude of the factor loadings both in the group factors and the cross-loadings. The study analyzes the recovery of factor loadings and focuses on the behavior of two groups of goodness-of-fit indices: Asymptotically unbiased estimators of fit indices (the RMSEA, the most widely used index; and the unbiased SRMR index, which is the only fit index formulated in a standardized metric with an associated statistical test of close fit), and biased estimators of fit indices commonly used in practice (the CFI, the GFI, and the SRMR). The purpose of the study was to examine the consequences of ignoring the cross-loadings on the estimation of the factor loadings and assess the sensitivity of the fit indices to detect model misspecification. Conditions regarding the characteristics of the model, such as the magnitude of the factor loadings or the sample size, were also manipulated to better understand the consequences of ignoring the cross-loadings and provide practical recommendations to researchers.

Concerning the recovery of factor loadings, our results indicated that ignoring the non-zero parameter cross-loadings of the bifactor model has a negative impact on the recovery of factor loadings, particularly when using small sample sizes (e.g., 200 or fewer observations). More specifically, the consequences of ignoring non-zero cross-loadings are that the loadings in the general factor are overestimated and those in the group factors are underestimated. These effects are more pronounced when the cross-loadings take larger values (0.20 or more) and the loadings in the group factors are smaller. These findings are congruent with previous research (Zhang et al., 2021a; Wei et al., 2022) and suggest that ignoring moderate and large cross-loadings and forcing them to take zero values will have a negative impact on parameter estimation. Our results of underestimation of the loadings in the group factors may explain the bias of overestimation of the loadings in the general factor and support that the phenomenon of factor collapse may have operated, such that the group factors improperly collapse onto the general factor. Thus, ignoring the cross-loadings in a confirmatory bifactor model may be problematic when they take moderate-to-large values and the sample size is small.

Concerning the goodness of fit, our results revealed that the biased fit indices (CFI, GFI, and SRMR) are not useful to detect model misspecification due to ignoring the non-zero cross-loadings, given that the use of Hu and Bentler’s (1999) cutoffs (CFI and GFI > 0.95, and SRMR <0.08) will lead us to conclude that all the tested conditions provide a close fit to the estimated model, even for the misspecified models. These results could be due to the fact that bifactor models better fit the data than other models (Morgan et al., 2015; Rodriguez et al., 2016). Concerning the unbiased fit indices (RMSEA and SRMR*_u_*), we found that the magnitude of the factor loadings affects those indices, such that the smaller the factor loading, the better the fit of the model. As explained above, this is the reliability paradox phenomenon, and these indices need to be corrected by considering the magnitude of the factor loadings. We evaluated Shi et al.’s (2018) correction for the SRMR*_u_* index based on the communality level (*R*^2^) and their cutoff criterion of ${\mathrm{SRMR}}_{u}/{\bar{R}}^{2}\leq 0.05$ to identify close-fitting models, and of ${\mathrm{SRMR}}_{u}/{\bar{R}}^{2}\leq 0.10$ to identify adequate-fitting models. Our results indicated that the ${\mathrm{SRMR}}_{u}/{\bar{R}}^{2}$ correction was accurate and, more importantly, could detect model misspecification due to ignoring the cross-loadings, regardless of sample size.

Based on our findings, we conclude that the biased fit indices (CFI, GFI, and SRMR) are not useful to detect model misspecification by constraining to zero the coefficients of the cross-loadings and, therefore, we do not recommend their use to assess the goodness of fit of confirmatory bifactor models. We recommend the use of unbiased fit indices and a confidence interval. When using RMSEA, researchers should be aware that this index comes in an unstandardized metric and will be more difficult to interpret. Moreover, the RMSEA will only detect misspecified bifactor models when the loadings in the group factors and the cross-loading take large values. We then recommend favoring the use of the unbiased SRMR index, which comes in a standardized metric, but also suggest applying Shi et al.’s (2018) correction based on the communality level to control for the effect of factor loading.

In conclusion, we recommend that the cross-loadings in the group factors be taken into account when assessing the factor pattern recovery in confirmatory models assuming a bifactor structure. This research has shown that ignoring the non-zero cross-loadings does not lead to the misfit of the model when SEM fit indexes and their cutoffs are used, but it will bias the parameter estimates and may lead to the group factors collapsing onto the general factor. Thus, we recommend researchers to model the cross-loadings in their bifactor models instead of forcing them to take zero values. Of course, it must be taken into account that cross-loadings must be modeled without affecting the identification of the model. We also recommend that researchers favor the use of the SRMR unbiased index with Shi et al.’s (2018) correction based on the magnitude of the factor loadings, as it is the only fit index that can detect model misspecification due to ignoring the cross-loadings in the bifactor confirmatory model (the unbiased SRMR index and its confidence intervals and tests of close fit are available in the lavaan package version 0.6–10 in R into the function *lavResiduals*).

As is the case with any simulation study, our results will hold only in conditions similar to those considered herein. Thus, future research should continue examining these effects under different study conditions. For instance, previous research argues that the problems of the cross-loadings can be overcome by using Exploratory SEM models (ESEM), which integrate EFA and CFA, allowing cross-loadings to be freely estimated rather than being constrained to zero (Asparouhov and Muthén, 2009); and Bayesian SEM (BSEM), where one can postulate cross-loadings not taking zero values and estimate them with reference to a prior distribution (Muthén and Asparouhov, 2012). Wei et al. (2022) found that these approaches performed similarly in the case of zero cross-loadings, but SEM performed worse as cross-loadings increased, ESEM exhibited unstable performance in conditions of small factor loadings, and the performance of BSEM depended on the accuracy of the priors for cross-loadings. Thus, future research could be directed to test the effects found here in such models and evaluate whether Shi et al.’s (2018) correction for the SRMR index based on communality level works reasonably well to detect model misspecification under these approaches. Another current line of research not considered here has to do with the performance of the augmentation strategy, consisting of adding an additional indicator that only loads onto the general factor to reduce the probability of nonidentification problems (Eid et al., 2017). Our simulation study did not considered such conditions but previous research encourages to adopt this strategy when analyzing data with bifactor modeling as it reduces estimation bias even with the presence of complex factor structures (Zhang et al., 2021a,b). Then, future research could also be directed to study the performance of the augmentation strategy when modeling non-zero cross-loadings. Finally, future studies should examine data other than those based on a normal distribution. For instance, further study could be directed to assess the impact of ignoring the cross-loadings in factor analysis of nominal data (Revuelta et al., 2020).

In closing, we hope that this research provides additional information to researchers to assist them when using bifactor models and conducting the difficult task of deciding whether or not to include the cross-loadings and select the appropriate index for assessing the goodness of fit of their models.

## Data availability statement

The raw data supporting the conclusions of this article will be made available by the authors, without undue reservation.

## Author contributions

CX contributed to the conception of the manuscript, the design and planning of the simulation study, the development of the R code for the data simulation, the statistical analysis and interpretation of results, and the drafting of the manuscript. JR contributed to the development of the R code for the data simulation and the revision of the manuscript. RC participated in the literature review, statistical analysis, and the revision of the manuscript. All authors contributed to the article and approved the submitted version.

## Funding

This work was supported by the grant no. PGC2018-093838-B-I00 from the Spanish Ministerio de Ciencia, Innovación y Universidades.

## Conflict of interest

The authors declare that the research was conducted in the absence of any commercial or financial relationships that could be construed as a potential conflict of interest.

## Publisher’s note

All claims expressed in this article are solely those of the authors and do not necessarily represent those of their affiliated organizations, or those of the publisher, the editors and the reviewers. Any product that may be evaluated in this article, or claim that may be made by its manufacturer, is not guaranteed or endorsed by the publisher.

## Footnotes

^1^Although unbiased estimators of the CFI have been developed (see Lai, 2019) but are not implemented in SEM software and will not be considered here.
