# Supplementary material for: What are the consequences of ignoring cross-loadings in bifactor models? A simulation study assessing parameter recovery and sensitivity of goodness-of-fit indices
Source: Front Psychol. 2022 Aug 18;13:923877. doi: 10.3389/fpsyg.2022.923877 (PMC9462382; doi:10.3389/fpsyg.2022.923877)
Supplement: Supplementary file 1 [file Data_Sheet_1.docx]

**Table S1.**

*Proportion of nonconvergent solutions and Heywood cases under the study conditions*

|  |  | NCONVER | | | | | | | | HEYWOOD | | | | | | | |
| --- | --- | --- | --- | --- | --- | --- | --- | --- | --- | --- | --- | --- | --- | --- | --- | --- | --- |
|  |  | Correct model | | | | Incorrect model | | | | Correct model | | | | Incorrect model | | | |
| *λ* | *c* | *N* = 100 | *N* = 200 | *N* = 500 | *N* = 1000 | *N* = 100 | *N* = 200 | *N* = 500 | *N* = 1000 | *N* = 100 | *N* = 200 | *N* = 500 | *N* = 1000 | *N* = 100 | *N* = 200 | *N* = 500 | *N* = 1000 |
| .15 | .05 | .06 | .05 | .05 | .05 | .03 | .04 | .04 | .05 | .12 | .10 | .05 | .04 | .14 | .10 | .05 | .04 |
|  | .10 | .06 | .05 | .05 | .05 | .03 | .04 | .05 | .05 | .12 | .09 | .06 | .03 | .12 | .10 | .07 | .03 |
|  | .20 | .06 | .05 | .05 | .04 | .03 | .03 | .04 | .04 | .12 | .09 | .04 | .02 | .13 | .09 | .06 | .03 |
|  | .30 | .04 | .03 | .03 | .02 | .03 | .02 | .03 | .02 | .10 | .07 | .03 | .01 | .11 | .07 | .04 | .02 |
|  | .40 | .04 | .02 | .01 | .00 | .02 | .02 | .02 | .00 | .08 | .06 | .02 | .00 | .10 | .06 | .02 | .01 |
| .30 | .05 | .03 | .02 | .01 | .00 | .02 | .01 | .01 | .00 | .07 | .05 | .02 | .00 | .07 | .05 | .02 | .00 |
|  | .10 | .03 | .02 | .01 | .00 | .02 | .02 | .01 | .00 | .08 | .05 | .02 | .00 | .07 | .05 | .02 | .00 |
|  | .20 | .03 | .02 | .01 | .00 | .02 | .01 | .01 | .00 | .07 | .05 | .02 | .00 | .08 | .05 | .02 | .00 |
|  | .30 | .03 | .02 | .00 | .00 | .01 | .01 | .00 | .00 | .07 | .05 | .01 | .00 | .09 | .05 | .01 | .00 |
|  | .40 | .02 | .01 | .00 | .00 | .01 | .01 | .00 | .00 | .06 | .02 | .00 | .00 | .07 | .04 | .01 | .00 |
| .50 | .05 | .01 | .00 | .00 | .00 | .00 | .00 | .00 | .00 | .03 | .01 | .00 | .00 | .02 | .01 | .00 | .00 |
|  | .10 | .01 | .00 | .00 | .00 | .00 | .00 | .00 | .00 | .03 | .01 | .00 | .00 | .04 | .01 | .00 | .00 |
|  | .20 | .01 | .00 | .00 | .00 | .00 | .00 | .00 | .00 | .03 | .01 | .00 | .00 | .04 | .01 | .00 | .00 |
|  | .30 | .01 | .00 | .00 | .00 | .00 | .00 | .00 | .00 | .03 | .01 | .00 | .00 | .04 | .01 | .00 | .00 |
|  | .40 | .01 | .00 | .00 | .00 | .00 | .00 | .00 | .00 | .02 | .01 | .00 | .00 | .03 | .02 | .00 | .00 |
| .60 | .05 | .01 | .00 | .00 | .00 | .00 | .00 | .00 | .00 | .02 | .01 | .00 | .00 | .02 | .01 | .00 | .00 |
|  | .10 | .01 | .00 | .00 | .00 | .00 | .00 | .00 | .00 | .02 | .01 | .00 | .00 | .02 | .01 | .00 | .00 |
|  | .20 | .01 | .00 | .00 | .00 | .00 | .00 | .00 | .00 | .03 | .01 | .00 | .00 | .01 | .01 | .00 | .00 |
|  | .30 | .01 | .00 | .00 | .00 | .00 | .00 | .00 | .00 | .02 | .01 | .00 | .00 | .02 | .01 | .00 | .00 |
|  | .40 | .01 | .00 | .00 | .00 | .00 | .00 | .00 | .00 | .02 | .00 | .00 | .00 | .02 | .00 | .00 | .00 |

*Note:* NCONVER = nonconvergent solutions, HEYWOOD = Heywood cases, *λ* = magnitude of loadings in the group factors, *c* = magnitude of cross-loadings, and *N* = sample size.

**Table S2.**

*Mean and standard deviations* (*in parenthesis*) *for the parameter recovery of the loadings in the general factor*

|  |  | RMSD_FGen | | | | | | | |
| --- | --- | --- | --- | --- | --- | --- | --- | --- | --- |
|  |  | Correct model | | | | Incorrect model | | | |
| *λ* | *c* | *N* = 100 | *N* = 200 | *N* = 500 | *N* = 1000 | *N* = 100 | *N* = 200 | *N* = 500 | *N* = 1000 |
| .15 | .05 | .12 (.03) | .09 (.02) | .05 (.01) | 04 (.01) | .12 (.03) | .09 (.02) | .05 (.01) | 04 (.01) |
|  | .10 | .12 (.03) | .09 (.02) | .05 (.01) | 04 (.01) | .12 (.03) | .09 (.02) | .05 (.01) | 04 (.01) |
|  | .20 | .12 (.03) | .09 (.02) | .05 (.01) | 04 (.01) | .12 (.03) | .09 (.02) | .05 (.01) | 04 (.01) |
|  | .30 | .12 (.03) | .09 (.02) | .06 (.01) | 04 (.01) | .12 (.03) | .09 (.02) | .06 (.01) | 04 (.01) |
|  | .40 | .12 (.03) | .09 (.02) | .05 (.01) | 04 (.01) | .12 (.03) | .09 (.02) | .06 (.01) | 04 (.01) |
| .30 | .05 | .12 (.03) | .09 (.02) | .06 (.01) | 04 (.01) | .12 (.03) | .09 (.02) | .06 (.01) | 04 (.01) |
|  | .10 | .13 (.03) | .09 (.02) | .06 (.01) | 04 (.01) | .13 (.03) | .09 (.02) | .06 (.01) | 05 (.01) |
|  | .20 | .13 (.03) | .09 (.02) | .06 (.01) | 04 (.01) | .13 (.03) | .09 (.02) | .06 (.01) | 04 (.01) |
|  | .30 | .13 (.03) | .10 (.02) | .06 (.01) | 04 (.01) | .13 (.03) | .09 (.02) | .06 (.01) | 04 (.01) |
|  | .40 | .13 (.03) | .10 (.02) | .06 (.01) | 04 (.01) | .13 (.03) | .10 (.02) | .07 (.02) | 06 (.01) |
| .50 | .05 | .15 (.04) | .11 (.03) | .07 (.02) | .05 (.01) | .15 (.04) | .11 (.03) | .07 (.02) | .05 (.01) |
|  | .10 | .15 (.04) | .11 (.03) | .07 (.02) | .05 (.01) | .14 (.04) | .11 (.03) | .07 (.02) | .05 (.01) |
|  | .20 | .15 (.04) | .11 (.03) | .07 (.02) | .05 (.01) | .15 (.04) | .11 (.03) | .07 (.02) | .05 (.01) |
|  | .30 | .15 (.04) | .11 (.03) | .07 (.02) | .05 (.01) | .15 (.04) | .11 (.03) | .08 (.02) | .07 (.01) |
|  | .40 | .15 (.04) | .11 (.03) | .07 (.02) | .05 (.01) | .16 (.04) | .12 (.03) | .09 (.02) | .08 (.01) |
| .60 | .05 | .17 (.05) | .12 (.03) | .08 (.02) | .05 (.01) | .17 (.05) | .12 (.03) | .08 (.02) | .05 (.01) |
|  | .10 | .17 (.05) | .13 (.03) | .08 (.02) | .05 (.01) | .17 (.05) | .13 (.03) | .08 (.02) | .05 (.01) |
|  | .20 | .17 (.05) | .13 (.03) | .08 (.02) | .05 (.01) | .17 (.04) | .13 (.03) | .08 (.02) | .06 (.01) |
|  | .30 | .17 (.05) | .13 (.03) | .08 (.02) | .05 (.01) | .18 (.04) | .13 (.03) | .09 (.02) | .08 (.01) |
|  | .40 | .18 (.05) | .13 (.04) | .07 (.02) | .05 (.01) | .19 (.06) | .13 (.04) | .08 (.02) | .06 (.02) |

*Note:* *λ* = magnitude of loadings in the group factors, *c* = magnitude of cross-loadings, and *N* = sample size.

**Table S3.**

*Mean and standard deviations* (*in parenthesis*) *for the parameter recovery of the loadings in the group factors and of the cross-loadings*

|  |  | RMSD_FGroup | | | | | | | | RMSD_Cross | | | |
| --- | --- | --- | --- | --- | --- | --- | --- | --- | --- | --- | --- | --- | --- |
|  |  | Correct model | | | | Incorrect model | | | | Correct model | | | |
| *λ* | *c* | *N* = 100 | *N* = 200 | *N* = 500 | *N* = 1000 | *N* = 100 | *N* = 200 | *N* = 500 | *N* = 1000 | *N* = 100 | *N* = 200 | *N* = 500 | *N* = 1000 |
| .15 | .05 | .26 (.04) | .21 (.04) | .17 (.04) | .14 (.03) | .26 (.04) | .21 (.03) | .17 (.03) | .14 (.03) | .21 (.08) | .17 (.07) | .14 (.06) | .12 (.04) |
|  | .10 | .25 (.04) | .22 (.04) | .17 (.03) | .14 (.04) | .25 (.04) | .22 (.03) | .17 (.03) | .14 (.04) | .20 (.08) | .17 (.07) | .13 (.05) | .12 (.05) |
|  | .20 | .26 (.04) | .21 (.04) | .17 (.04) | .14 (.04) | .25 (.04) | .22 (.03) | .18 (.03) | .15 (.03) | .21 (.09) | .18 (.07) | .15 (.07) | .13 (.06) |
|  | .30 | .26 (.04) | .22 (.04) | .17 (.04) | .14 (.04) | .26 (.04) | .23 (.03) | .19 (.03) | .17 (.03) | .23 (.10) | .20 (.09) | .17 (.08) | .14 (.07) |
|  | .40 | .26 (.05) | .22 (.05) | .16 (.05) | .12 (.04) | .27 (.04) | .24 (.03) | .21 (.03) | .20 (.03) | .27 (.12) | .22 (.10) | .17 (.10) | .11 (.08) |
| .30 | .05 | .24 (.06) | .20 (.06) | .14 (.05) | .09 (.03) | .25 (.05) | .20 (.04) | .14 (.04) | .09 (.03) | .21 (.08) | .17 (.07) | .12 (.05) | .10 (.04) |
|  | .10 | .25 (.06) | .20 (.05) | .14 (.05) | .10 (.03) | .26 (.05) | .20 (.05) | .14 (.04) | .10 (.03) | .20 (.08) | .17 (.07) | .13 (.05) | .10 (.04) |
|  | .20 | .25 (.06) | .20 (.06) | .14 (.05) | .09 (.04) | .26 (.05) | .20 (.05) | .14 (.04) | .11 (.03) | .20 (.08) | .17 (.07) | .13 (.06) | .10 (.05) |
|  | .30 | .25 (.06) | .20 (.06) | .13 (.05) | .09 (.03) | .27 (.05) | .22 (.05) | .16 (.04) | .13 (.04) | .22 (.10) | .18 (.08) | .12 (.06) | .08 (.05) |
|  | .40 | .25 (.06) | .20 (.06) | .12 (.04) | .08 (.02) | .28 (.05) | .24 (.05) | .19 (.04) | .16 (.04) | .23 (.11) | .18 (.10) | .11 (.07) | .07 (.04) |
| .50 | .05 | .24 (.08) | .16 (.06) | .09 (.03) | .06 (.02) | .25 (.06) | .16 (.05) | .09 (.02) | .06 (.01) | .19 (.08) | .15 (.06) | .10 (.04) | .07 (.03) |
|  | .10 | .23 (.08) | .16 (.06) | .09 (.03) | .06 (.02) | .25 (.06) | .16 (.05) | .09 (.03) | .07 (.01) | .19 (.08) | .15 (.06) | .10 (.04) | .07 (.03) |
|  | .20 | .23 (.08) | .16 (.07) | .09 (.03) | .06 (.02) | .25 (.07) | .16 (.05) | .10 (.03) | .08 (.02) | .20 (.08) | .15 (.07) | .10 (.05) | .07 (.03) |
|  | .30 | .24 (.08) | .16 (.06) | .09 (.03) | .06 (.01) | .26 (.07) | .18 (.05) | .12 (.03) | .09 (.02) | .20 (.09) | .15 (.08) | .09 (.05) | .06 (.03) |
|  | .40 | .23 (.08) | .16 (.06) | .09 (.02) | .06 (.01) | .27 (.07) | .20 (.06) | .14 (.04) | .11 (.02) | .20 (.10) | .14 (.08) | .08 (.04) | .06 (.03) |
| .60 | .05 | .23 (.10) | .15 (.07) | .09 (.03) | .06 (.01) | .25 (.08) | .15 (.05) | .09 (.02) | .06 (.01) | .18 (.08) | .14 (.06) | .09 (.04) | .06 (.03) |
|  | .10 | .23 (.10) | .15 (.07) | .09 (.03) | .06 (.01) | .25 (.08) | .15 (.05) | .09 (.02) | .06 (.01) | .19 (.08) | .14 (.06) | .09 (.04) | .06 (.03) |
|  | .20 | .23 (.10) | .15 (.07) | .08 (.02) | .06 (.01) | .25 (.08) | .16 (.05) | .10 (.02) | .07 (.01) | .19 (.08) | .14 (.06) | .08 (.04) | .06 (.03) |
|  | .30 | .23 (.10) | .15 (.06) | .08 (.03) | .06 (.01) | .26 (.08) | .17 (.06) | .11 (.03) | .09 (.02) | .19 (.09) | .14 (.07) | .08 (.04) | .06 (.02) |
|  | .40 | .23 (.10) | .15 (.06) | .08 (.02) | .06 (.01) | .27 (.08) | .19 (.06) | .13 (.03) | .11 (.02) | .19 (.10) | .13 (.07) | .08 (.03) | .05 (.02) |

*Note:* *λ* = magnitude of loadings in the group factors, *c* = magnitude of cross-loadings, and *N* = sample size.

**Table S4.**

*Mean and standard deviations* (*in parenthesis*) *for the RMSEA and the SRMR indices*

| *RMSEA* | | | | | | | | | | | *SRMR* | | | | | | | | |
| --- | --- | --- | --- | --- | --- | --- | --- | --- | --- | --- | --- | --- | --- | --- | --- | --- | --- | --- | --- |
|  |  | Correct model | | | | Incorrect model | | | | | Correct model | | | | Incorrect model | | | | |
| *λ* | *c* | *N* = 100 | *N* = 200 | *N* = 500 | *N* = 1000 | *pop* | *N* = 100 | *N* = 200 | *N* = 500 | *N* = 1000 | *N* = 100 | *N* = 200 | *N* = 500 | *N* = 1000 | *pop* | *N* = 100 | *N* = 200 | *N* = 500 | *N* = 1000 |
| .15 | .05 | .03 (.02) | .01 (.02) | .01 (.01) | .00 (.01) | .07 | .03 (.02) | .01 (.02) | .01 (.01) | .00 (.01) | .05 (.01) | .04 (.00) | .02 (.00) | .02 (.00) | .04 | .05 (.01) | .04 (.00) | .02 (.00) | .02 (.00) |
|  | .10 | .03 (.02) | .01 (.02) | .01 (.01) | .00 (.01) | .07 | .03 (.02) | .01 (.02) | .01 (.01) | .01 (.01) | .05 (.01) | .04 (.00) | .02 (.00) | .02 (.00) | .04 | .05 (.01) | .04 (.00) | .02 (.00) | .02 (.00) |
|  | .20 | .02 (.02) | .01 (.02) | .01 (.01) | .00 (.01) | .07 | .03 (.02) | .01 (.02) | .01 (.01) | .01 (.01) | .05 (.01) | .04 (.00) | .02 (.00) | .02 (.00) | .04 | .05 (.01) | .04 (.00) | .02 (.00) | .02 (.00) |
|  | .30 | .02 (.02) | .01 (.02) | .01 (.01) | .00 (.01) | .07 | .03 (.02) | .01 (.02) | .01 (.01) | .01 (.01) | .05 (.01) | .04 (.00) | .02 (.00) | .02 (.00) | .04 | .05 (.01) | .04 (.00) | .02 (.00) | .02 (.00) |
|  | .40 | .02 (.02) | .01 (.02) | .01 (.01) | .00 (.01) | .07 | .03 (.03) | .02 (.02) | .01 (.01) | .01 (.01) | .05 (.01) | .04 (.00) | .02 (.00) | .02 (.00) | .04 | .05 (.01) | .04 (.00) | .02 (.00) | .02 (.00) |
| .30 | .05 | .02 (.02) | .01 (.02) | .01 (.01) | .01 (.01) | .03 | .02 (.02) | .01 (.02) | .01 (.01) | .01 (.01) | .05 (.01) | .04 (.00) | .02 (.00) | .02 (.00) | .02 | .05 (.01) | .04 (.00) | .02 (.00) | .02 (.00) |
|  | .10 | .02 (.02) | .01 (.02) | .01 (.01) | .01 (.01) | .03 | .03 (.02) | .01 (.02) | .01 (.01) | .01 (.01) | .05 (.01) | .04 (.00) | .02 (.00) | .02 (.00) | .02 | .05 (.01) | .04 (.00) | .02 (.00) | .02 (.00) |
|  | .20 | .02 (.02) | .01 (.02) | .01 (.01) | .01 (.01) | .04 | .02 (.02) | .02 (.02) | .01 (.01) | .01 (.01) | .05 (.01) | .04 (.00) | .02 (.00) | .02 (.00) | .02 | .05 (.01) | .04 (.00) | .02 (.00) | .02 (.00) |
|  | .30 | .02 (.02) | .01 (.02) | .01 (.01) | .01 (.01) | .05 | .03 (.03) | .02 (.02) | .02 (.01) | .02 (.01) | .05 (.01) | .04 (.00) | .02 (.00) | .02 (.00) | .03 | .05 (.01) | .04 (.00) | .03 (.00) | .02 (.00) |
|  | .40 | .02 (.02) | .01 (.02) | .01 (.01) | .01 (.01) | .07 | .03 (.03) | .03 (.02) | .03 (.01) | .03 (.01) | .05 (.01) | .04 (.00) | .02 (.00) | .02 (.00) | .03 | .05 (.01) | .04 (.00) | .03 (.00) | .02 (.00) |
| .50 | .05 | .02 (.02) | .01 (.02) | .01 (.01) | .01 (.01) | .01 | .03 (.02) | .01 (.02) | .01 (.01) | .01 (.01) | .05 (.01) | .03 (.00) | .02 (.00) | .01 (.00) | .01 | .05 (.01) | .04 (.00) | .02 (.00) | .02 (.00) |
|  | .10 | .02 (.02) | .01 (.02) | .01 (.01) | .01 (.01) | .02 | .03 (.02) | .02 (.02) | .01 (.01) | .01 (.01) | .05 (.01) | .03 (.00) | .02 (.00) | .01 (.00) | .01 | .05 (.01) | .04 (.00) | .02 (.00) | .02 (.00) |
|  | .20 | .02 (.02) | .01 (.02) | .01 (.01) | .01 (.01) | .05 | .03 (.03) | .03 (.02) | .02 (.01) | .02 (.01) | .05 (.01) | .03 (.00) | .02 (.00) | .01 (.00) | .02 | .05 (.01) | .04 (.00) | .03 (.00) | .02 (.00) |
|  | .30 | .02 (.02) | .01 (.02) | .01 (.01) | .01 (.01) | .07 | .03 (.03) | .03 (.02) | .03 (.01) | .03 (.01) | .05 (.01) | .03 (.00) | .02 (.00) | .02 (.00) | .04 | .05 (.01) | .04 (.01) | .03 (.00) | .02 (.00) |
|  | .40 | .02 (.02) | .01 (.02) | .01 (.01) | .01 (.01) | .11 | .04 (.03) | .04 (.02) | .04 (.01) | .04 (.00) | .05 (.01) | .03 (.00) | .02 (.00) | .02 (.00) | .05 | .06 (.01) | .04 (.01) | .03 (.00) | .03 (.00) |
| .60 | .05 | .02 (.02) | .01 (.02) | .01 (.01) | .01 (.01) | .01 | .02 (.02) | .02 (.02) | .01 (.01) | .01 (.01) | .05 (.01) | .03 (.00) | .02 (.00) | .01 (.00) | .01 | .05 (.01) | .03 (.00) | .02 (.00) | .02 (.00) |
|  | .10 | .02 (.02) | .01 (.02) | .01 (.01) | .01 (.01) | .03 | .03 (.02) | .02 (.02) | .01 (.01) | .01 (.01) | .05 (.01) | .03 (.00) | .02 (.00) | .01 (.00) | .01 | .05 (.01) | .04 (.00) | .02 (.00) | .02 (.00) |
|  | .20 | .02 (.02) | .01 (.02) | .01 (.01) | .01 (.01) | .06 | .03 (.02) | .02 (.02) | .02 (.01) | .02 (.01) | .05 (.01) | .03 (.00) | .02 (.00) | .01 (.00) | .03 | .05 (.01) | .04 (.00) | .03 (.00) | .02 (.00) |
|  | .30 | .02 (.02) | .01 (.02) | .01 (.01) | .01 (.01) | .10 | .04 (.03) | .03 (.02) | .03 (.01) | .04 (.01) | .05 (.01) | .03 (.00) | .02 (.00) | .01 (.00) | .04 | .06 (.01) | .04 (.00) | .03 (.00) | .03 (.00) |
|  | .40 | .02 (.02) | .01 (.02) | .01 (.01) | .01 (.01) | .14 | .05 (.03) | .05 (.02) | .05 (.01) | .05 (.00) | .05 (.01) | .03 (.00) | .02 (.00) | .01 (.00) | .05 | .06 (.01) | .05 (.00) | .04 (.00) | .03 (.00) |

*Note:* *RMSEA* = root mean squared error of approximation, *SRMR* = standardized root mean squared residual, *λ* = magnitude of loadings in the group factors, *c* = magnitude of cross-loadings, *N* = sample size, and *pop* = population index value.

**Table S5.**

*Mean and standard deviations* (*in parenthesis*) *for the CFI and the GFI indices*

| *CFI* | | | | | | | | | | | *GFI* | | | | | | | | |
| --- | --- | --- | --- | --- | --- | --- | --- | --- | --- | --- | --- | --- | --- | --- | --- | --- | --- | --- | --- |
|  |  | Correct model | | | | Incorrect model | | | | | Correct model | | | | Incorrect model | | | | |
| *λ* | *c* | *N* = 100 | *N* = 200 | *N* = 500 | *N* = 1000 | *pop* | *N* = 100 | *N* = 200 | *N* = 500 | *N* = 1000 | *N* = 100 | *N* = 200 | *N* = 500 | *N* = 1000 | *pop* | *N* = 100 | *N* = 200 | *N* = 500 | *N* = 1000 |
| .15 | .05 | .98 (.02) | .99 (.01) | 1.00 (.0) | 1.00 (.0) | .94 | .98 (.02) | .99 (.01) | 1.00 (.0) | 1.00 (.0) | .92 (.01) | .96 (.01) | .98 (.00) | .99 (.00) | .97 | .92 (.01) | .96 (.01) | .98 (.00) | .99 (.00) |
|  | .10 | .98 (.02) | .99 (.01) | 1.00 (.0) | 1.00 (.0) | .94 | .98 (.02) | .99 (.01) | 1.00 (.0) | 1.00 (.0) | .92 (.01) | .96 (.01) | .98 (.00) | .99 (.00) | .97 | .92 (.01) | .96 (.01) | .98 (.00) | .99 (.00) |
|  | .20 | .98 (.02) | .99 (.01) | 1.00 (.0) | 1.00 (.0) | .94 | .98 (.02) | .99 (.01) | 1.00 (.0) | 1.00 (.0) | .92 (.01) | .96 (.01) | .98 (.00) | .99 (.00) | .97 | .92 (.01) | .96 (.01) | .98 (.00) | .99 (.00) |
|  | .30 | .98 (.02) | .99 (.01) | 1.00 (.0) | 1.00 (.0) | .94 | .98 (.02) | .99 (.01) | 1.00 (.0) | 1.00 (.0) | .92 (.01) | .96 (.01) | .98 (.00) | .99 (.00) | .97 | .92 (.01) | .96 (.01) | .98 (.00) | .99 (.00) |
|  | .40 | .99 (.02) | 1.00 (.0) | 1.00 (.0) | 1.00 (.0) | .94 | .99 (.02) | .99 (.01) | 1.00 (.0) | 1.00 (.0) | .92 (.01) | .96 (.01) | .98 (.00) | .99 (.00) | .97 | .92 (.01) | .96 (.01) | .98 (.00) | .99 (.00) |
| .30 | .05 | .98 (.02) | .99 (.01) | 1.00 (.0) | 1.00 (.0) | .99 | .98 (.02) | .99 (.01) | 1.00 (.0) | 1.00 (.0) | .92 (.01) | .96 (.01) | .98 (.00) | .99 (.00) | .99 | .92 (.01) | .96 (.01) | .98 (.00) | .99 (.00) |
|  | .10 | .98 (.02) | .99 (.01) | 1.00 (.0) | 1.00 (.0) | .99 | .98 (.02) | .99 (.01) | 1.00 (.0) | 1.00 (.0) | .92 (.01) | .96 (.01) | .98 (.00) | .99 (.00) | .99 | .92 (.01) | .96 (.01) | .98 (.00) | .99 (.00) |
|  | .20 | .99 (.02) | .99 (.01) | 1.00 (.0) | 1.00 (.0) | .98 | .98 (.02) | .99 (.01) | 1.00 (.0) | 1.00 (.0) | .92 (.01) | .96 (.01) | .98 (.00) | .99 (.00) | .99 | .92 (.01) | .96 (.01) | .98 (.00) | .99 (.00) |
|  | .30 | .99 (.02) | .99 (.01) | 1.00 (.0) | 1.00 (.0) | .97 | .98 (.02) | .99 (.01) | .99 (.01) | .99 (.00) | .92 (.01) | .96 (.01) | .98 (.00) | .99 (.00) | .98 | .92 (.01) | .95 (.01) | .98 (.00) | .99 (.00) |
|  | .40 | .99 (.02) | .99 (.01) | 1.00 (.0) | 1.00 (.0) | .96 | .98 (.03) | .99 (.01) | .99 (.01) | .99 (.00) | .92 (.01) | .96 (.01) | .98 (.00) | .99 (.00) | .97 | .91 (.01) | .95 (.01) | .97 (.00) | .98 (.00) |
| .50 | .05 | .99 (.02) | .99 (.01) | 1.00 (.0) | 1.00 (.0) | .99 | .98 (.02) | .99 (.01) | 1.00 (.0) | 1.00 (.0) | .92 (.01) | .96 (.01) | .98 (.00) | .99 (.00) | .99 | .92 (.01) | .96 (.01) | .98 (.00) | .99 (.00) |
|  | .10 | .99 (.02) | .99 (.01) | 1.00 (.0) | 1.00 (.0) | .99 | .98 (.02) | .99 (.01) | 1.00 (.0) | 1.00 (.0) | .92 (.01) | .96 (.01) | .98 (.00) | .99 (.00) | .99 | .92 (.01) | .96 (.01) | .98 (.00) | .99 (.00) |
|  | .20 | .99 (.02) | .99 (.01) | 1.00 (.0) | 1.00 (.0) | .98 | .98 (.02) | .99 (.01) | .99 (.01) | .99 (.00) | .92 (.01) | .96 (.01) | .98 (.00) | .99 (.00) | .99 | .92 (.01) | .95 (.01) | .98 (.00) | .99 (.00) |
|  | .30 | .99 (.02) | .99 (.01) | 1.00 (.0) | 1.00 (.0) | .97 | .98 (.02) | .98 (.01) | .99 (.01) | .99 (.00) | .92 (.01) | .96 (.01) | .98 (.00) | .99 (.00) | .96 | .91 (.01) | .95 (.01) | .97 (.00) | .98 (.00) |
|  | .40 | .99 (.02) | .99 (.01) | 1.00 (.0) | 1.00 (.0) | .93 | .97 (.03) | .98 (.02) | .98 (.01) | .98 (.00) | .92 (.01) | .96 (.01) | .98 (.00) | .99 (.00) | .93 | .91 (.01) | .94 (.01) | .97 (.00) | .97 (.00) |
| .60 | .05 | .99 (.02) | .99 (.01) | 1.00 (.0) | 1.00 (.0) | .99 | .99 (.02) | .99 (.01) | 1.00 (.0) | 1.00 (.0) | .92 (.01) | .96 (.01) | .98 (.00) | .99 (.00) | .99 | .92 (.01) | .96 (.01) | .98 (.00) | .99 (.00) |
|  | .10 | .99 (.02) | .99 (.01) | 1.00 (.0) | 1.00 (.0) | .99 | .98 (.02) | .99 (.01) | .99 (.01) | .99 (.00) | .92 (.01) | .96 (.01) | .98 (.00) | .99 (.00) | .99 | .92 (.01) | .96 (.01) | .98 (.00) | .99 (.00) |
|  | .20 | .99 (.02) | .99 (.01) | 1.00 (.0) | 1.00 (.0) | .98 | .98 (.02) | .99 (.01) | .99 (.01) | .99 (.00) | .92 (.01) | .96 (.01) | .98 (.00) | .99 (.00) | .98 | .92 (.01) | .95 (.01) | .98 (.00) | .99 (.00) |
|  | .30 | .99 (.02) | .99 (.01) | 1.00 (.0) | 1.00 (.0) | .96 | .98 (.02) | .98 (.01) | .99 (.01) | .98 (.00) | .92 (.01) | .96 (.01) | .98 (.00) | .99 (.00) | .94 | .91 (.01) | .95 (.01) | .97 (.00) | .98 (.00) |
|  | .40 | .99 (.02) | 1.00 (.0) | 1.00 (.0) | 1.00 (.0) | .91 | .97 (.03) | .97 (.01) | .97 (.01) | .97 (.01) | .92 (.01) | .96 (.01) | .98 (.00) | .99 (.00) | .87 | .90 (.01) | .94 (.01) | .96 (.00) | .97 (.00) |

*Note:* *CFI* = comparative fit index, *GFI* = goodness-of-fit index, *λ* = magnitude of loadings in the group factors, *c* = magnitude of cross-loadings, *N* = sample size, and *pop* = population index value.

**Table S6.**

*Mean and standard deviations* (*in parenthesis*) *for the SRMR_u_ index and its correction* $SRMR_{u}/\bar{R}^{2}$

|  |  |  | *SRMR_u_* | | | | | | | | |  | | | | | | | |
| --- | --- | --- | --- | --- | --- | --- | --- | --- | --- | --- | --- | --- | --- | --- | --- | --- | --- | --- | --- |
|  |  |  | Correct model | | | | Incorrect model | | | | | Correct model | | | | Incorrect model | | | |
| *λ* | *c* | $\bar{R}^{2}$ | *N* = 100 | *N* = 200 | *N* = 500 | *N* = 1000 | *pop* | *N* = 100 | *N* = 200 | *N* = 500 | *N* = 1000 | *N* = 100 | *N* = 200 | *N* = 500 | *N* = 1000 | *N* = 100 | *N* = 200 | *N* = 500 | *N* = 1000 |
| .15 | .05 | .38 | .02 (.01) | .01 (.01) | .00 (.00) | .00 (.00) | .04 | .04 (.01) | .04 (.01) | .04 (.01) | .03 (.00) | .04 (.03) | .02 (.02) | .01 (.01) | .01 (.01) | .12 (.02) | .11 (.02) | .10 (.02) | .08 (.01) |
|  | .10 | .39 | .02 (.01) | .01 (.01) | .00 (.00) | .00 (.00) | .04 | .03 (.01) | .03 (.00) | .03 (.01) | .02 (.00) | .04 (.03) | .02 (.02) | .01 (.01) | .01 (.01) | .08 (.03) | .08 (.01) | .07 (.01) | .06 (.01) |
|  | .20 | .42 | .02 (.01) | .01 (.01) | .00 (.00) | .00 (.00) | .04 | .03 (.01) | .03 (.01) | .03 (.00) | .02 (.00) | .04 (.03) | .02 (.02) | .01 (.01) | .01 (.01) | .07 (.02) | .07 (.02) | .07 (.01) | .06 (.01) |
|  | .30 | .47 | .01 (.01) | .01 (.01) | .00 (.00) | .00 (.00) | .04 | .02 (.01) | .02 (.01) | .01 (.00) | .01 (.00) | .03 (.03) | .02 (.02) | .01 (.01) | .01 (.01) | .05 (.02) | .05 (.02) | .05 (.01) | .04 (.01) |
|  | .40 | .54 | .01 (.01) | .01 (.01) | .00 (.00) | .00 (.00) | .04 | .02 (.01) | .02 (.00) | .02 (.01) | .01 (.00) | .03 (.02) | .01 (.01) | .01 (.01) | .01 (.01) | .05 (.02) | .05 (.01) | .04 (.01) | .03 (.01) |
| .30 | .05 | .45 | .01 (.01) | .01 (.01) | .00 (.00) | .00 (.00) | .02 | .03 (.01) | .02 (.01) | .01 (.01) | .01 (.01) | .03 (.03) | .02 (.02) | .01 (.01) | .01 (.01) | .07 (.02) | .06 (.02) | .05 (.01) | .03 (.01) |
|  | .10 | .46 | .01 (.01) | .01 (.01) | .00 (.00) | .00 (.00) | .02 | .03 (.01) | .03 (.01) | .03 (.01) | .01 (.00) | .03 (.03) | .02 (.02) | .01 (.01) | .01 (.01) | .07 (.02) | .07 (.02) | .06 (.01) | .03 (.01) |
|  | .20 | .49 | .01 (.01) | .01 (.01) | .00 (.00) | .00 (.00) | .02 | .03 (.01) | .02 (.01) | .02 (.01) | .01 (.01) | .03 (.03) | .02 (.01) | .01 (.01) | .01 (.01) | .07 (.02) | .05 (.02) | .04 (.01) | .03 (.01) |
|  | .30 | .54 | .01 (.01) | .01 (.01) | .00 (.00) | .00 (.00) | .03 | .02 (.01) | .02 (.01) | .02 (.01) | .01 (.00) | .02 (.02) | .01 (.01) | .01 (.01) | .01 (.01) | .05 (.02) | .05 (.02) | .04 (.01) | .03 (.01) |
|  | .40 | .61 | .01 (.01) | .01 (.01) | .00 (.00) | .00 (.00) | .03 | .02 (.01) | .02 (.01) | .02 (.01) | .01 (.00) | .02 (.02) | .01 (.01) | .01 (.01) | .01 (.01) | .05 (.02) | .04 (.02) | .03 (.01) | .02 (.01) |
| .50 | .05 | .61 | .01 (.01) | .01 (.01) | .00 (.00) | .00 (.00) | .01 | .03 (.01) | .02 (.01) | .02 (.00) | .01 (.00) | .02 (.02) | .01 (.01) | .01 (.01) | .01 (.01) | .05 (.02) | .05 (.01) | .05 (.01) | .03 (.01) |
|  | .10 | .62 | .01 (.01) | .01 (.01) | .00 (.00) | .00 (.00) | .01 | .03 (.01) | .02 (.01) | .02 (.01) | .01 (.00) | .02 (.02) | .01 (.01) | .01 (.01) | .01 (.01) | .05 (.02) | .05 (.01) | .05 (.01) | .03 (.01) |
|  | .20 | .65 | .01 (.01) | .01 (.01) | .00 (.00) | .00 (.00) | .02 | .03 (.01) | .02 (.01) | .02 (.01) | .01 (.00) | .02 (.02) | .01 (.01) | .01 (.01) | .01 (.00) | .05 (.02) | .04 (.02) | .04 (.01) | .03 (.01) |
|  | .30 | .70 | .01 (.01) | .01 (.01) | .00 (.00) | .00 (.00) | .04 | .03 (.01) | .02 (.01) | .02 (.01) | .01 (.01) | .02 (.02) | .01 (.01) | .01 (.01) | .00 (.00) | .04 (.02) | .04 (.02) | .04 (.01) | .04 (.01) |
|  | .40 | .77 | .01 (.01) | .01 (.01) | .00 (.00) | .00 (.00) | .05 | .03 (.01) | .02 (.01) | .02 (.00) | .01 (.00) | .01 (.01) | .01 (.01) | .01 (.01) | .00 (.00) | .04 (.02) | .04 (.01) | .04 (.01) | .03 (.01) |
| .60 | .05 | .72 | .01 (.01) | .01 (.01) | .00 (.00) | .00 (.00) | .01 | .03 (.01) | .03 (.01) | .03 (.00) | .02 (.00) | .02 (.02) | .01 (.01) | .01 (.01) | .00 (.00) | .05 (.02) | .05 (.02) | .05 (.01) | .04 (.01) |
|  | .10 | .73 | .01 (.01) | .01 (.01) | .00 (.00) | .00 (.00) | .01 | .03 (.01) | .03 (.01) | .03 (.01) | .03 (.00) | .02 (.02) | .01 (.01) | .01 (.01) | .00 (.00) | .05 (.02) | .05 (.02) | .04 (.01) | .04 (.01) |
|  | .20 | .76 | .01 (.01) | .01 (.01) | .00 (.00) | .00 (.00) | .03 | .04 (.01) | .04 (.01) | .03 (.01) | .03 (.00) | .01 (.01) | .01 (.01) | .01 (.01) | .00 (.00) | .06 (.02) | .06 (.01) | .05 (.01) | .04 (.01) |
|  | .30 | .81 | .01 (.01) | .01 (.01) | .00 (.00) | .00 (.00) | .04 | .04 (.02) | .03 (.01) | .03 (.00) | .03 (.01) | .01 (.01) | .01 (.01) | .01 (.01) | .00 (.00) | .05 (.02) | .05 (.01) | .05 (.01) | .04 (.01) |
|  | .40 | .88 | .01 (.01) | .01 (.01) | .00 (.00) | .00 (.00) | .05 | .04 (.02) | .04 (.01) | .04 (.01) | .04 (.01) | .01 (.01) | .01 (.01) | .01 (.01) | .00 (.00) | .05 (.02) | .05 (.02) | .05 (.01) | .05 (.01) |

*Note:* *SRMR_u_* = unbiased SRMR index, *λ* = magnitude of loadings in the group factors, *c* = magnitude of cross-loadings, $\bar{R}^{2}$= communality level, *N* = sample size, and *pop* = population index value. $SRMR_{u}/\bar{R}^{2}$ is the Shi et al’s (2018) correction for the *SRMR_u_* index based on communality.
